# Clinical translation of theranostic radiopharmaceuticals: Current regulatory status and recent examples

**DOI:** 10.1002/jlcr.3712

**Published:** 2019-03-21

**Authors:** Petra Kolenc Peitl, Christine Rangger, Piotr Garnuszek, Renata Mikolajczak, Alicja Hubalewska‐Dydejczyk, Theodosia Maina, Paola Erba, Clemens Decristoforo

**Affiliations:** ^1^ Deparment of Nuclear Medicine University Medical Centre Ljubljana Ljubljana Slovenia; ^2^ Department of Nuclear Medicine Medical University Innsbruck Innsbruck Austria; ^3^ Radioisotope Centre POLATOM National Centre for Nuclear Research Otwock Poland; ^4^ Chair and Department of Endocrinology Jagiellonian University Medical College Krakow Poland; ^5^ Molecular Radiopharmacy INRASTES, NCSR “Demokritos” Athens Greece; ^6^ Nuclear Medicine Department of Translational Research and New Technologies in Medicine University of Pisa and Azienda Ospedaliero‐Universitaria Pisana Pisa Italy

**Keywords:** ^111^In‐CP04, clinical trial, peptide radiopharmaceuticals, regulatory framework, theranostics nuclear medicine

## Abstract

With the development of ever more radiopharmaceuticals suitable for theranostic applications, translation of novel compounds from the preclinical stage towards clinical application becomes a bottleneck for the advances in Nuclear Medicine. This review article summarizes the current regulatory framework for clinical trials with radiopharmaceuticals in the European Union, provides a general overview of the documentation required, and addresses quality, safety, and clinical aspects to be considered. By using a recent successful example of translating a theranostic peptide radioligand, namely ^111^In‐CP04, which targets receptors expressed in medullary thyroid carcinoma, the pathway from the preclinical development over establishing the required pharmaceutical documentation to designing and submitting a clinical trial is reviewed. Details regarding preclinical data, generation of the documentation, and final successful application are described. This article should provide an insight in an ever more complex process to bring innovations in the field of radiopharmaceuticals into patients.

## 1 INTRODUCTION

The concept of combining imaging properties with the therapeutic potential of decaying radionuclides in an integrated theranostic concept has received a tremendous spin within the last years.^1^ This has in the first place emerged from the long awaited registration of ^177^Lu‐DOTATATE (Lutathera). It received marketing authorization in Europe and the US in 2018,^2^ together with its “companion diagnostic” a kit formulation for Ga‐68 labelling as Edotreotide (DOTATOC) in the EU (Somakit TOC) and DOTATATE in the US (NetSpot), being the first peptide‐based theranostic for wider clinical use. A second stimulus originated from the introduction of PSMA targeting ligands for diagnosis and therapy in prostate cancer.^3^ [^68^Ga]Ga ‐PSMA ‐11^4^ has proven to be a highly sensitive and specific marker for early detection and localization of prostate lesions in the biochemical recurrence stage and other clinical indications. Based on the promising imaging results, the development of DOTA‐based ligands for labelling with Lu‐177, PSMA 617,^5^ and PSMA I&T^6^ have stimulated the research in this field with ever more improved ligands being developed, exemplified by the impressive clinical results of Ac‐225 labelled PSMA 617, selected as the radiopharmaceutical of the year 2017.^7^

Research in expanding this concept to other theranostic applications in oncology and also other clinical fields is impressively vital. Numerous peptide analogues targeting G‐protein coupled receptors overexpressed on tumor cells have been developed. Many of these compounds have now reached a stage for clinical translation showing optimized preclinical profiles, including metabolic stability, antagonism, and high binding affinity for their cognate receptor. Representative examples are somatostatin and gastrin‐releasing peptide receptor antagonists, new cholecystokinin 2 receptor (CCK_2_R) targeting ligands, or exendin analogues.^8^ Not only peptides, but also antigen targeting compounds, such as single domain antibody fragments,^9^ or small molecules for pretargeting strategies, eg, based on biorthogonal click chemistry,^10^ have the potential for a number of clinical applications. Another promising field is the utilization of small molecules with high affinity to cancer associated targets, such as fibroblast activating peptide inhibitors (FAPI), recently showing impressive targeting in various tumors.^11^

Today, drug development is driven by pharmaceutical industry designing, initiating, and financing controlled clinical trials from Phase I to Phase III with the final aim of commercialization, ie, achieving marketing authorization. From a historical perspective, the success of the clinical translation of the theranostic concept with radiopharmaceuticals was originally based on academic developments and initiatives. Many of them were introduced in several European countries with a regulatory framework that allowed the use of novel concepts both for diagnosis and therapy outside the strict clinical trial pathway,^12^ based on approvals on a local level without involvement of national drug regulatory agencies. Over the past years, most European countries have set increased requirements, allowing the use of novel diagnostic and in particular therapeutic radiopharmaceuticals only after formal approval of a prospective clinical trial application by the national drug authorities.

**Figure 1:**
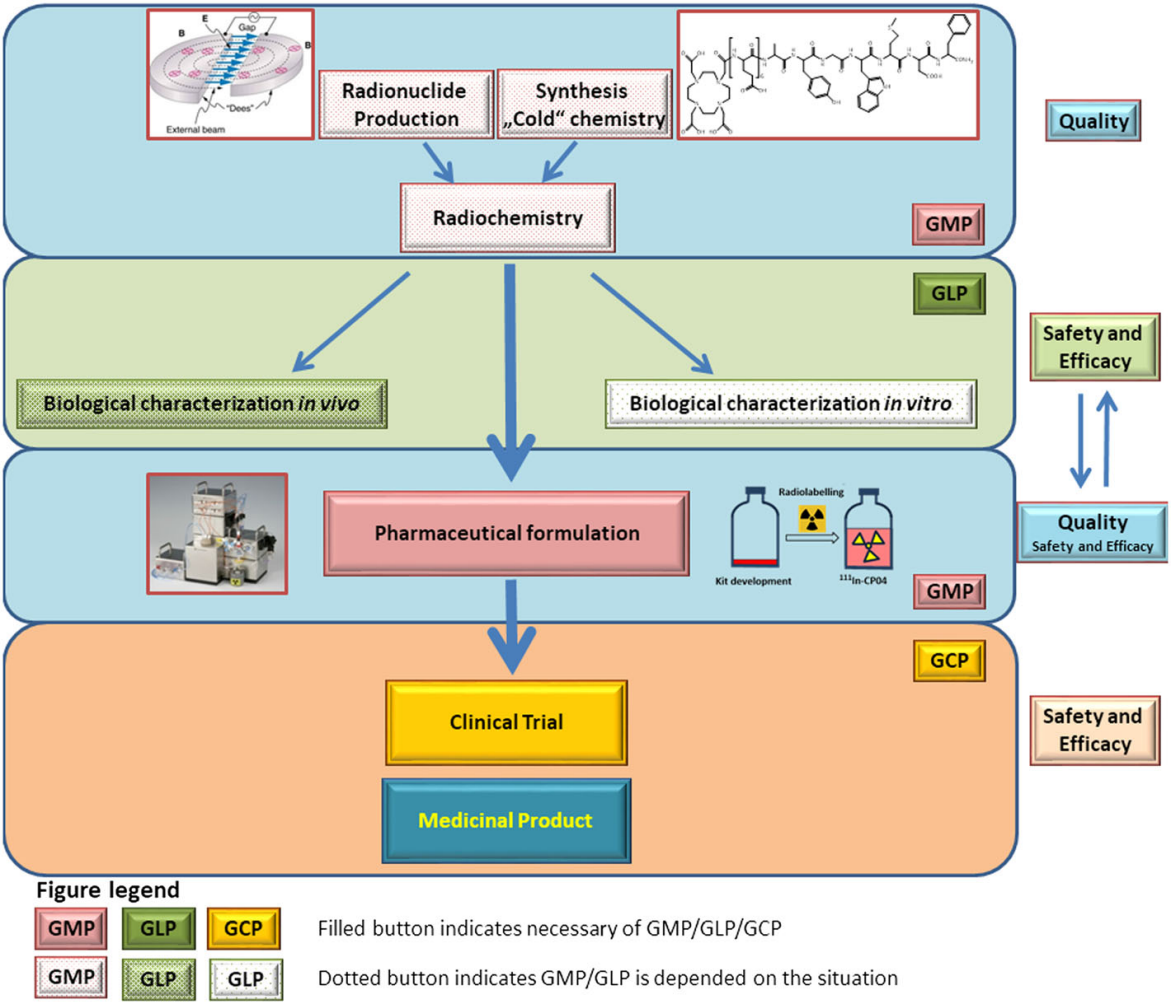
Path of a new radiopharmaceutical to the patient. *Upper blue panel*. In case of radiopharmaceuticals, one has to consider the radioactive part (radionuclide) and “cold” ligand and possibly radiolabelled entity as a drug substance. *Green panel*. Biological characterization *in vitro* and *in vivo* constitutes the essential part for assessment of preclinical safety and efficacy of the potential radiopharmaceutical (and/or “cold” ligand and radionuclide). Some studies need to be done under GLP conditions (ie, toxicity studies). *Middle blue panel*. Before applying for a clinical trial, a radiopharmaceutical has to be properly formulated following GMP principles. The formulated drug is compared with the unformulated one in respect to efficacy and/or safety. *Orange panel*. After submission of the required documentation (see Figure 2), the clinical trial can start, following GCP. If clinical safety and efficacy are proven, a clinical trial can potentially lead to a medicinal product (radiopharmaceutical) with a marketing authorization

The overall pathway of a novel radiopharmaceutical from preclinical development to a final medicinal product is outlined in Figure 1. This short review will, in more detail, address the current regulatory environment for clinical trials, critical data required to ensure quality and safety of novel theranostic compounds for clinical translation, examples of clinical translations, and finally recent developments and outlook for the translation of theranostic concepts with radiopharmaceuticals.

## 2 CURRENT REGULATORY FRAMEWORK EU

**Figure 2:**
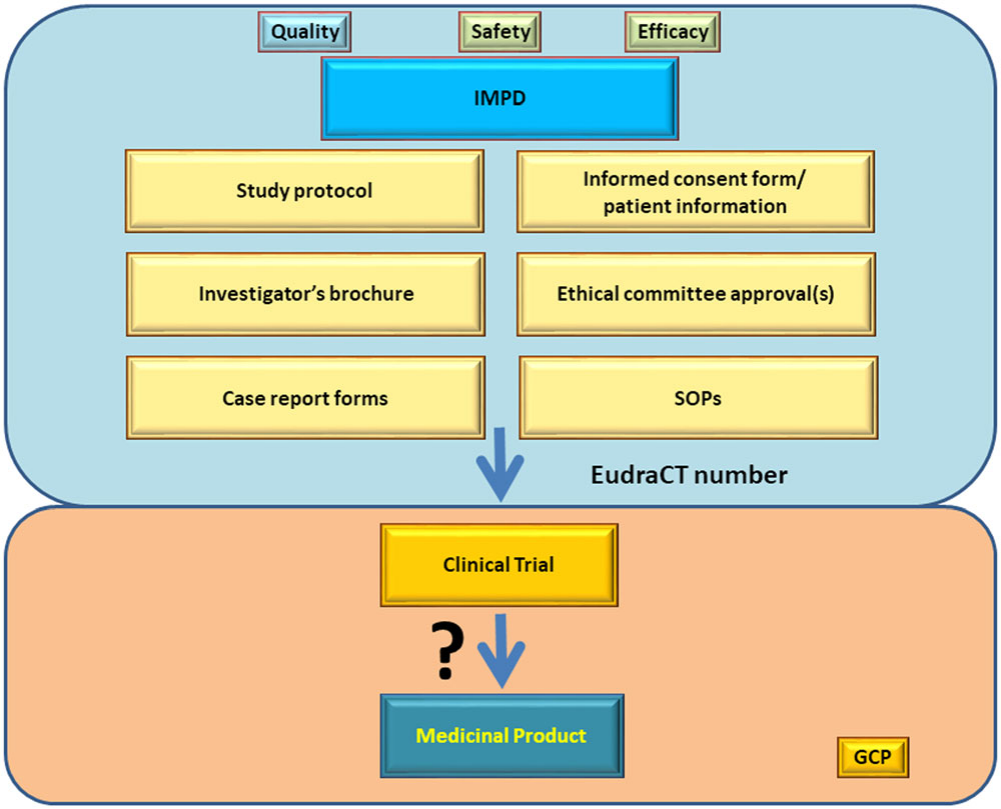
Submission process scheme. *Blue panel*. Investigational medicinal product dossier (IMPD) containing all information obtained in the upper three panels of Figure 1 (quality aspects of radionuclide, ligand and final radiopharmaceutical formulation production/preparation; safety and efficacy aspects from preclinical animal studies) together with some other key documents like study protocol, investigator's brochure, or informed consent forms to various SOPs enables submission of the trial under designated EudraCT number. *Orange panel*. After approval, the clinical trial can be initiated and could potentially lead to a medicinal product (radiopharmaceutical) with a marketing authorization

Several articles summarize the regulatory framework for radiopharmaceuticals in Europe^13^ and specifically the regulation on early‐phase clinical trials.^12^, ^14^ Radiopharmaceuticals are defined as medicinal products, also including kits, radionuclide precursors, and generators. By this, the use of a new radiopharmaceutical in patients or volunteers should be conducted within a clinical trial as a so‐called Investigational Medicinal Product (IMP). The current “Clinical Trials Directive” defines the requirement of authorization of manufacturing an IMP, which includes applying Good Manufacturing Practices (GMP). Within this regulatory framework, also Good Clinical Practices (GCP) for conducting clinical trials are mandatory, stating, eg, responsibilities, requirements, and structure of clinical trials (ICH E6 GCP).^15^ The application process as outlined in Figure 2 follows clearly defined rules. The clinical trial application has to be approved by both a (national) ethical committee and the (national) competent authority within defined timelines. It took many years until the full scope of the clinical trial regulation, in particular Directive 2001/20, showed its full impact in particular in relation to radiopharmaceuticals. The number of clinical trial applications declined, and therefore the European Commission initiated a revision process, which resulted in a new Regulation (EU) No 536/2014. The new Regulation aims at simplifying and speeding up approval of clinical trials by a central electronic portal, where all applications have to be submitted and reviewed within very strict timelines. This portal, however, is not yet functional delaying the implementation of the new Regulation, which is not expected before end of 2019. Interestingly, the new Regulation clearly recognized specific requirements for radiopharmaceuticals.^16^ It includes exemptions for diagnostic radiopharmaceuticals from the need for manufacturing authorization and GMP and introduces reduced requirements for the labelling of radiopharmaceuticals as IMPs.

The US pathway for submission and approval of clinical trials follows a different route with a central agency, the FDA, being responsible for reviewing. Differences between Europe and the US regarding radiopharmaceuticals have recently been addressed.^17^ However, similar documents are required to complete a clinical trial application dossier, which are addressed in the following chapters of this review.

## 3 DOCUMENTATION

A documentation package for a clinical trial application includes both information on the IMP as well as on the conduct of the clinical trial itself. The main information of the radiopharmaceutical to be used in the trial are contained in the Investigational Medicinal Product Dossier (IMPD). It is important to understand that the information in the IMPD has to be given in a standardized way, which is based on the so‐called Common Technical Dossier (CTD) format, which is also used in applications for marketing authorization. The documents differentiate the chemical and pharmaceutical information package (Quality) from the nonclinical and clinical safety and efficacy information, including toxicology and pharmacology of the new radiopharmaceutical. This differentiation in particular is important for radiopharmaceuticals; therefore, specific guidance has been released by the EANM to provide information on how an IMPD for a radiopharmaceutical can be designed and is a very useful reference when coping with this task.^18^

## 4 QUALITY ASPECTS

The first part of the IMPD addresses chemical and pharmaceutical properties covering the quality of a new radiopharmaceutical. This part of the information is divided in the drug substance and drug product information^19^; the required data are summarized in Table 1. In classical drugs, the drug substance describes the active pharmaceutical ingredient (API), its production, characterization, and analysis. The drug product part contains all information about the formulated IMP, its production, characterization, analysis, and stability. In case of radiopharmaceuticals, this differentiation is often challenging as they are prepared from predefined radioactive and nonradioactive (“chemical”) precursors and the radiopharmaceutical is never isolated as it is usually the case with conventional medicines.

**Table 1:** Quality data required for translation of a radiopharmaceutical

Data	Purpose	Example of CP04
Drug substance		
*Precursor*	Production and analytical data, specifications, stability	1,4,7,10‐tetraazacyclododecane‐N,N′,N″,N‴‐tetraacetic acid (DOTA)‐dGlu‐dGlu‐dGlu‐dGlu‐dGlu‐dGlu‐Ala‐Tyr‐Gly‐Trp‐Met‐Asp‐Phe‐NH_2_, CP04, MG48
*Radionuclide*	Production and analytical data, specifications, stability	^111^InCl_3_, indium (^111^In) chloride, DRN4901
*Reference materials*		CP04 CRS
*Drug product*		
*Formulation*	Development, composition, manufacturing process	Kit composition:
		*CP04.TFA salt*
		*L‐ascorbic acid*
		*Gentisic acid*
		*L‐methionine*
		*Sodium hydroxide*
		*Nitrogen*
Excipients	Controls, stability, producer, CoA	Ph. Eur., current valid edition:
		*L‐ascorbic acid*
		*L‐methionine*
		*Sodium hydroxide*
		*Water for injection*
		Nonpharmacopoeial excipients:
		Gentisic acid (2,5‐dihydroxybenzoic acid)
		Nitrogen protective gas in vials and used during manufacture for purging.
Analytical control	Validation, impurities, specifications	1. Test for pH, sterility, and endotoxins follow Ph. Eur.
		2. Validation of HPLC method for CP04 assay and radiochemical purity ^111^In‐CP04
		3. Impurities: CP04 oxidized; [^111^In]‐CP04 ox (≤5%), ^111^In free (≤5%)
*Stability*	Shelf life, storage	• The shelf life of the CP04 kits for radiopharmaceutical preparation: 12 months
		• Stability of the radiolabelled ^111^In‐CP04 preparation: 4 h after radiolabelling
		• Storage conditions: CP04 cold kits should be stored in a refrigerator (−2°C to 8°C)
		• Radiolabelled preparation (kit after radiolabelling) should be stored at temperatures from 15°C to 25°C

Therefore, drug authorities expect a great deal of information on the quality of radionuclide and chemical precursors, as they are considered as API, in the drug substance part. Especially if precursors are not removed by purification steps, which is usually the case in theranostic applications with radiometals, the chemical precursor, eg, a peptide targeting a receptor, needs to be prepared according to GMP by an authorized manufacturer, resulting in one of the major costs arising in translational activities.

Besides describing the preparation process of the radiopharmaceutical, its development, and its validation, a great piece of information deals with the definition of release criteria, analytical procedures, and especially their validation. An important reference for the definition of specification and selection of analytical methods is the European Pharmacopeia (Ph. Eur.).^20^ Here, not only specific monographs for certain radiopharmaceuticals can be found, but also more general guidance in general chapters and monographs on radiopharmaceuticals, chemical precursors, and general analytical methods that may apply. Besides describing the test methods specifications, also stability data both of the final radiopharmaceutical as well as the precursors are of high importance in the summary of the quality data of new radiopharmaceuticals. In the part describing the “Investigational Product under Test” (or the “drug product”), it is also important to characterize all excipients including their quality used in the preparation.

## 5 SAFETY ASPECTS

In the second part of the IMPD, information on the safety and efficacy of the IMP should be provided. This includes both nonclinical as well as clinical information. At this moment, no dedicated guidance is available from the regulatory bodies on how to present this documentation. In case of a new radiopharmaceutical, the earlier the clinical phase, the more information on safety will be expected, whereas in later phases requirements shift towards efficacy data.

Especially for novel radiopharmaceuticals, no or very limited clinical information for the radiopharmaceutical will be available; however, it is recommended to provide an overview of related compounds and their available clinical data (in view of safety and efficacy). For example, if a new peptide is to be evaluated, clinical data on the same class, targeting the same receptor, will be available and will be important to finally contribute to an appropriate risk vs benefit analysis.

The expected nonclinical safety data are summarized in Table 2. Nonclinical pharmacology has to provide information on pharmacokinetics of the radiopharmaceutical. This should include *in vitro* tests for stability and other parameters, such as lipophilicity or protein binding, but mainly *in vivo* studies in healthy animals and disease models. This data has to be complemented with information on the target interaction, usually *in vitro* studies on binding affinity, functional profile (agonistic or antagonistic), and, if relevant, specificity and ‐off target effects (eg, receptor [subtype] specificity). Again, *in vivo* targeting data need to be provided. In the specific case of theranostics and translation into a clinical therapy setting, data on the therapeutic efficacy in animal models should be considered as well.

**Table 2:** Nonclinical safety data required for translation of a radiopharmaceutical

Data	Purpose	Example of CP04
Pharmacology	In vitro binding affinity	Binding affinities were evaluated in surgically resected human tumor tissues. IC_50_: CP04 = 1.8 ± 1.2, [^nat^In]‐CP04 = 2.5 ± 1.4 nM
	Internalization rate	AR42J cell lines; after 4‐h incubation, 37°C, 5% CO_2_; expressed in percentage of injected activity per million cells: 8.9 ± 1.3
Pharmacokinetics	In vitro stability, protein binding, lipophilicity	Stability in human serum: (175 ± 71) h
		Ca. 10%
		logD = −3.9
	In vivo biodistribution/imaging	1. Lewis male rats implanted subcutaneously AR42J tumor cells: (1.24 ± 0.43) % IA/g, tumor/kidney = 1.12, (4 h p.i.)
		2. SCID mice bearing A431‐CCK2R(+/−) xenografts: (9.24 ± 1.35)) % IA/g, 1.66 (4 h p.i.)
Toxicity	Dosimetry study (biodistribution)	Expected equivalent human absorbed doses of ^111^In‐CP04 based on two models for translating mouse to human data would be 0.045 mSv/MBq (9.9 mSv at planned dose of 220 MBq of ^111^In‐CP04).
	Acute toxicity	Mice; LD^50^ (mouse): Greater than 178.5 μg/kg body weight
	Extended single dose toxicity	Rats; 89 μg/kg can be considered the NOAEL

For radiopharmaceuticals, nonclinical safety data also have to include dosimetry investigations. These are an essential part of the safety and toxicity evaluation of a radiopharmaceutical as the application of radioactivity is the dominating risk for teratogenicity, genotoxicity, and cancerogenicity, which in conventional medicines is evaluated in separate, dedicated tests. In general, the nonclinical safety studies required for pharmaceuticals are described in a dedicated ICH guideline M3(R2).^21^ This guideline also describes the “microdosing‐concept,” which is often applied to radiopharmaceuticals for the evaluation of the toxicity of the nonradioactive component. This allows reducing the toxicity tests mainly to an extended single dose toxicity study. However, this concept only covers administrations below 100 μg of drug substance and is not dedicated to the specific case of a radiopharmaceutical. In addition, the required extended single dose toxicity study has to be performed under Good Laboratory Practice (GLP), which is another major cost driver in the clinical translation of radiopharmaceuticals. The limitations of the current guidelines and practice are discussed in a recent position paper of the EANM,^22^ and European Medicines Agency (EMA) has recently announced to release a more specific guidance for the nonclinical (safety) evaluation of radiopharmaceuticals,^23^ which should clarify, harmonize, and hopefully simplify this process. It should be mentioned that for theranostic radiopharmaceuticals, the safety evaluation for the therapeutic application may require additional toxicity tests, in particular in later phases of the clinical development. This is for example demanded by the FDA in a recent guidance document^24^ to evaluate late radiation toxicity effects of therapeutic radiopharmaceuticals.

## 6 CLINICAL ASPECTS

The conduct of a clinical trial has to follow strict regulations that are not only defined in EU legislation, but are also implemented in the national law of the member state concerned. Besides these legally binding instruments for clinical trials, a very important document to mention is the Declaration of Helsinki which offers a set of ethical guidelines for physicians and other participants in medical research.^25^

All these legally binding and ethical standards have to be taken into consideration when planning a clinical trial. As a first step, a sponsor has to be declared. This sponsor can be for example an individual, a government agency, a pharmaceutical (drug) company, or an academic unit. The sponsor takes responsibility for managing and financing of the study which can be rather cost ‐intensive. In addition, the sponsor and researcher need to sign a mandatory insurance contract for civil responsibility for potential damages caused in the course of the clinical trial. As a next step, the clinical trial has to be approved by the national competent authority and the local ethical committee of the participating country. For the approval, different documents are needed which have to be adjusted for each country. The most important document is the clinical trial application form (CTA) together with the EudraCT number. These documents are generated using the European Union Drug Regulating Authorities Clinical Trials (EudraCT) database system (https://eudract.ema.europa.eu/) where every clinical trial, which will be conducted within Europe, has to be announced to the European Medicines Agency (EMA). The EudraCT number is specific for a certain trial and must be used in all correspondences with authorities and ethics committee. Another essential part for the approval is the study protocol. It describes the objective(s), design, methodology (criteria for patient inclusion/exclusion), statistical considerations, and the organization of a clinical trial and usually contains also information about the background and the scientific justification for the clinical trial.

Two other necessary documents are the investigator's brochure (IB) and the already described IMPD. While the IB is a compilation of clinical and nonclinical data on the IMP, the IMPD provides further high‐level information on the quality and production of the IMP, the toxicological and pharmacological examinations, and overall risk and benefit assessment of the IMP.

A patient‐related crucial document is the informed consent form (ICF). In the ICF, the patient declares that he/she has been informed about all risks involved, probable consequences of the trial, and also available alternatives. Together with the ICF, a patient information leaflet is provided where all trial relevant procedures are explained in a language understandable by a layman. In the ICF, the patient can also find the policy number of the mandatory for clinical trials insurance.

For the correct documentation and evaluation of the trial, a case report form (CRF) is vital. In the CRF, specific data (eg, blood tests, demographic data, vital signs) from each patient are collected throughout the trial. The blinded (all patient relevant information is removed) data is then processed by the sponsor and help to test hypotheses or answer research questions.

Additionally, all participating facilities/centres have to provide standard operating procedures (SOP), specific manuals, agreements between the sponsor and the trial site, CVs of the principal investigator, and information about the supporting staff.

In accordance with ICH E6 GCP, also trial monitoring is important.^26^ The monitor, designated by the sponsor, has to ensure that the well‐being and rights of the study patients are protected and has to verify that all collected data is complete and accurate. Furthermore, the monitor has to control that all conducted procedures are in compliance with the approved study protocol, with GCP, and with the pertinent laws and regulations of the member state.

## 7 EXAMPLE OF CP04

In the following, we will provide some insight in the translational process of a novel minigastrin analogue targeting medullary thyroid cancer (MTC). Diagnosis and treatment of metastatic MTC still remain a challenging clinical task. The CCK_2_R is one of the most promising targets for personalized diagnosis and treatment being overexpressed in MTC with very high density and >90% incidence. Within a COST action (BM0607), a number of different radiolabelled minigastrin analogues targeting CCK_2_R were tested preclinically and compared in a concerted effort to develop a new radiopharmaceutical for diagnosis and treatment of MTC. CP04, 1,4,7,10‐tetraazacyclododecane‐N,N′,N″,N‴‐tetraacetic acid (DOTA)‐dGlu‐dGlu‐dGlu‐dGlu‐dGlu‐dGlu‐Ala‐Tyr‐Gly‐Trp‐Met‐Asp‐Phe‐NH_2_, showed most promising characteristics, such as high stability and CCK_2_R affinity, specific and persistent tumor uptake, and low kidney retention in animal models. This analogue was selected for further clinical evaluation within an EU‐funded project (ERA‐NET GRAN‐T‐MTC).

### 7.1 Quality aspects

Initial studies on radiochemistry and consecutive pharmaceutical development were designed in order to collect data necessary for preparation of Part 2 of the IMPD for the IMP ^111^In‐CP04. Because the clinical trial was planned multicentric, it was necessary that CP04 is labelled with ^111^In on site at the clinics with high radiolabelling yield in a convenient and reproducible way. For these reasons, the nonradioactive component of the investigational product, the synthetic peptide‐conjugate CP04, was prepared in the form of a radiopharmaceutical kit. The kit contained CP04 and excipients needed to label it successfully with the radioactive component of the drug substance—^111^In. Indium (^111^In) chloride with the status of medicinal product (marketing authorization DRN 4901, Mallinckrodt Medical B.V.) was used in all centres.

CP04 was synthesized by Fmoc solid phase peptide synthesis (SPPS). It was manufactured and controlled according to cGMP by a pharmaceutical manufacturer of APIs (piCHEM GmbH, Graz, Austria). The manufacturer provided a description of manufacturing process and process controls, list of starting materials used for synthesis, and analysis of potential impurities in the final peptide product. Quality specification for CP04 as API was established based on general requirements for synthetic peptides and Ph. Eur. requirements for substances for pharmaceutical use. The identity of peptide was confirmed by mass spectrometry and analysis of amino acid residues (AAA), peptide purity was assessed by HPLC methods, assay of the net peptide was based on AAA results, residual solvents (acetonitrile and DMF), trifluoroacetate, and water contents were determined by gas chromatography (GC) methods, and bacterial endotoxins content by the LAL method. All analytical methods were briefly described with their validation status. The manufacturer's certificates for the drug substance components, ie, ^111^In and CP04 were presented (release and retest).

Preliminary experiments were performed in order to establish reliable conditions for radiopharmaceutical kit manufacture. Over 20 kit batches were freeze‐dried under aseptic conditions to develop a clinically suitable kit formulation. The kits with two doses of peptide were prepared: one containing 10 μg and another containing 50 μg of CP04. As excipients, L‐methionine, ascorbic acid, nitrogen of pharmaceutical grade as well as gentisic acid and sodium hydroxide of ultrapure quality were used. The immediate containers were 2‐mL clear borosilicate glass vials from SCHOTT (StandardLine, FIOLAX, Müllheim, Germany) corresponding to Ph. Eur. Type I, with chlorobutyl rubber stoppers. All these materials were fully characterized in the IMPD. Long‐term stability studies were performed at 2°C to 8°C and accelerated stability testing at 25°C and supported a high stability of the kits for 24 months when stored at 2°C to 8°C.

Quality specification of CP04 radiopharmaceutical kits was based on general Ph. Eur. monograph “Radiopharmaceutical preparations”: appearance of the kit content before and after dissolution in 1 mL of water, identification and assay of CP04 by HPLC using reference standard of CP04 CRS, radiochemical purity limits for ^111^In‐CP04 (≥90%), unbound ^111^In^3+^ (≤5%), and oxidized form of ^111^In‐CP04ox (≤5%) were checked by HPLC. Safety tests such as sterility and bacterial endotoxins content were specified. All analytical methods were fully validated.

Data on the chemical and pharmaceutical development and characterization of a freeze‐dried kit formulation for radiolabelling of the radiopharmaceutical precursor CP04 with ^111^In (chapter 2.1.P of the CTD) have been recently presented.^27^

### 7.2 Safety/efficacy data generated (pharmacology, toxicology)

Safety and efficacy information was also required to generate a suitable IMPD file for ^111^In‐CP04 to submit the Clinical Trials. This included pharmacology, toxicology, and dosimetry data that had to be retrieved from preclinical studies *in vitro* and in suitable animal models, which were summarized recently.^28^

The binding affinity of CP04 and ^111/nat^In‐CP04 for the CCK_2_R and the in vitro metabolic stability of ^111^In‐CP04 were previously reported.^29^, ^30^ New experiments in healthy mice revealed a fair *in vivo* metabolic stability of ^111^In‐CP04, and data was included in the IMPD. Previous biodistribution of ^111^In‐CP04 in A431‐CCK_2_R‐expressing tumors xenografts in mice^31^ showed high uptake and retention in tumor tissue, low kidney accumulation, and fast clearance from blood and background tissues. Further data retrieved in the same mouse model demonstrated an indistinguishable pharmacokinetic profile of ^111^In‐CP04 prepared from the freeze‐dried kit and by “wet‐labelling,” strongly supporting the efficacy and suitability of the freeze‐dried kit formulation for use in the clinical trial. Next, the plasma expander gelofusine was co‐injected in mice, inducing a significant reduction of renal uptake of ^111^In‐CP04. This strategy was considered as an option to follow in the clinical trial protocol.

In view of the fact that during previous small‐scale clinical studies conducted in MTC patients other CCK_2_R‐targeting peptide analogues elicited transient adverse effects, the toxicity of the CP04 precursor was extensively tested in two animal species. First, an acute intravenous toxicity study for CP04 based on OECD Guideline 423 (“Acute Oral Toxicity—Acute Toxic Class Method,” adopted on 17 December 2001) was conducted in mice (Harlan Laboratories Study #D04314), providing an LD_50_ > 178.5 μg/kg body weight for mice. Next, an extended acute single dose toxicity study (Harlan Laboratories Study #S47364) was conducted in rats according to the microdosing concept as described in European Medicines Agency,^21^ providing a no‐observed‐adverse‐effect‐level (NOAEL) of 89 μg/kg body weight for rats. It should be noted that the human equivalent dose (HED) calculated for this NOAEL value was 14.4 μg/kg (HED (μg/kg) = NOAEL × rat km/human km = 89 μg/kg × 6/37= 14.4 μg/kg). By taking into account a safety factor of 10, the maximum recommended starting dose (MRSD) for a first‐in‐human clinical trial would be 1.4 μg/kg. According to the CP04 clinical trial protocol (EudraCT No 2015‐000,805‐38), only adults with confirmed metastatic MTC were planned to be included in the study. Subjects were planned to be intravenously injected with either a lower peptide dose of 10 μg/patient or a higher peptide dose of 50 μg/patient. It should be noted that both doses are well below the MRSD. The abovementioned data was also included in the IMPD.

Dosimetric calculations derived from animal data constitute an integral part radiation‐associated risk assessment for new radiopharmaceuticals. Such data were retrieved and included in the IMPD based on biodistribution data of ^111^In‐CP04 in healthy mice for a 30‐min to 72‐h period post injection, showing the highest absorbed doses for kidneys. By employing two different dosimetric scaling options to translate mouse data to the human situation,^32^ almost identical values were obtained.^28^ The effective activity dose predicted for ^111^In‐CP04 in the clinical trial was less than 10 mSv (9.9 mSv/220 MBq), well below that of the licensed product OctreoScan in SST2R imaging with SPECT (effective dose of 26 mSv/220 MBq according to SPC).

### 7.3 Clinical documents and submission process

The central document for the clinical trial was the Clinical Trial Protocol. The trial was designed as a Phase I multicentre trial to include patients with progressive/metastatic nonoperable histologically proven MTC; details of the study design can be found in Erba et al.^33^ The main objectives were (1) to establish the safety of i.v. administration of a high peptide amount of ^111^In‐CP04 potentially also suitable for therapy and (2) to assess the tracer's biodistribution and to determine critical organs. The evaluation of the potential of CCK_2_R scintigraphy to detect cancer lesions for low (10 μg) and high peptide amount (50 μg) and the decrease of kidney dose after co‐administration of gelofusine (nephroprotective agent) were also included. Appropriate exclusion criteria were defined, and patient numbers were selected based on biostatistical calculation. A study agreement was elaborated, clarifying responsibilities of all participating centres, principle investigators, and sponsor (university of Pisa, Italy). CRFs were established electronically to allow central evaluation of study data from multiple participating centres, various SOPs and forms were defined for all participating centres, and an EudraCT number (2015‐000805‐38) for the study was obtained.

All study centres additionally had to design ICFs and fill in specific application forms for ethical committees and competent authorities based on national requirements. Also, insurance and study monitoring were included in the responsibilities of each participating centre. To ensure standardized data generation and to harmonize SPECT acquisition, phantom measurement protocols were centrally defined and locally implemented. In a similar way, radiolabelling and QC procedures were verified in each centre based on a standardized protocol before initiation of the study. Finally, four clinical centres in four different countries received national approval for the trial. Between 2015 and 2018, in total 16 patients were successfully enrolled in the study. Safety and dosimetry of ^111^In‐CP04 were established, and MTC metastases could be detected with high sensitivity^33^ which opened the way for a continuing therapeutic trial following a similar pathway with the corresponding ^177^Lu‐counterpart to implement the theranostic approach.

## 8 CONCLUSION

The translation of new theranostic radiopharmaceuticals requires clinical trials, which are heavily regulated within the EU, but also in most countries worldwide. In Europe, regulators have recognized the need for facilitation of clinical trials. This could be partly realized within the new Clinical Trial Regulation providing simplification especially for multicentric and multinational trials. However, still major hurdles exist that are related to high costs of toxicity testing and complying with GMP, but also with the numbers of documents to be generated for submission of a trial. Professional organizations such as the EANM are trying to give guidance for this, and also regulatory bodies such as EMA have recognized the need for specific instructions on particular topics. These efforts should be acknowledged, as they provide great help to address all requirements. Also, local support from clinical trial units at universities can support the translation process. Joining forces within multinational project applications and more interdisciplinary projects will be necessary to realize the full potential of the increasing number of developments for theranostic applications. The GRAN‐T‐MTC project described in this article is an excellent example for a successful translation in the theranostic setting. The speed of innovation and high number of developments in the future require scientists to focus on those applications with highest impact on patient care, and there exists the potential to improve currently available diagnostic and therapeutic applications. The recent high interest of pharmaceutical companies in theranostic applications with radiopharmaceuticals is expected to boost the availability of a broad spectrum of clinically useful agents in the years to come.
